# Chronic pain after traumatic brain injury: a collaborative care approach

**DOI:** 10.3389/fresc.2024.1398856

**Published:** 2024-08-26

**Authors:** Mary C. Curran, Sylvia Lucas, Jesse R. Fann, Jennifer M. Zumsteg, Jeanne M. Hoffman

**Affiliations:** ^1^ Department of Rehabilitation Medicine, University of Washington School of Medicine, Seattle, WA, United States; ^2^ Department of Neurological Surgery, University of Washington School of Medicine, Seattle, WA, United States; ^3^ Department of Neurology, University of Washington School of Medicine, Seattle, WA, United States; ^4^ Department of Psychiatry and Behavioral Sciences, University of Washington School of Medicine, Seattle, WA, United States; ^5^ Valley Medical Center, Seattle, WA, United States

**Keywords:** traumatic brain injury, chronic pain, collaborative care, intervention, cognitive behavioral therapy

## Abstract

**Introduction:**

Chronic pain is common after traumatic brain injury (TBI), frequently limits daily activities, and is associated with negative outcomes such as decreased community participation. Despite the negative impact of chronic pain, few people with TBI receive effective treatment. This paper describes a collaborative care (CC) intervention, TBI Care, adapted specifically to treat chronic pain in people living with TBI, emphasizing expert clinician input, cognitive behavioral therapy (CBT) techniques, and other non-pharmacological approaches for decreasing pain interference.

**Methods:**

79 participants engaged in the CC intervention from two academic medical rehabilitation clinics with weekly assessments of pain intensity, interference, and medication use. Participant feedback on the intervention was gathered by interview with the care manager (CM) at the last treatment session and/or booster session. Provider feedback was gathered by a confidential survey post intervention.

**Results:**

Ninety percent of participants received at least 11 of the target 12 sessions with a care manager (CM), the majority occurring over the phone. Participants endorsed an average of 7 pain locations. All participants received pain education, skills in self-monitoring, goal setting/behavioral activation and relaxation training. Pain interference scores (impact on activity and enjoyment), tracked weekly by the CM, significantly decreased across sessions. 89% of participants received recommendations for CBT skills, 65% received referrals for additional treatments targeting pain interference, and 43% received care coordination. 75% of participants reported 6 or more medications/supplements at both the first and last session, with changes recommended primarily for headache treatment. Feedback from participants and providers was positive.

**Discussion:**

TBI Care, a novel patient-centered CC approach, was flexibly delivered, tailored to the needs of those living with TBI and chronic pain, with a high level of participant engagement, and satisfaction among participants and providers. This approach, prioritizing pain self-management strategies and other non-pharmacological approaches, along with optimizing pharmacological treatment, led to significant reductions in self-reported pain interference and intensity during the intervention. Using a CC model in TBI is feasible and successfully improved access to evidence-based treatments for chronic pain as well as outcomes for pain interference and intensity.

**Clinical Trial Registration:**

ClinicalTrials.gov, identifier NCT03523923.

## Introduction

Traumatic brain injury (TBI) is common in the United States with approximately 2.9 million new injuries reported every year (1). Pain is a frequent complaint after TBI of all severities, with more than half of patients reporting pain (2, 3). Chronic pain, defined as pain experienced over at least 3 months for more than half the days, is prevalent in approximately 51% of civilian samples (2). Chronic pain is also associated with poor outcomes such as decreased community participation (4), quality of life, and interference with daily activities (5, 6). A recent pain survey conducted with 3,804 TBI Model Systems participants, found that chronic pain affects approximately 60% of those living with TBI from 1 to 30 years post injury (3). Chronic pain after TBI is often associated with, or exacerbated by, other comorbid conditions including depression, insomnia, and anxiety (7–9).

Despite the significant impact of pain on quality of life, interference with daily activities and interaction with comorbid conditions (10), many patients do not receive effective treatment for these often-disabling conditions. Barriers to effective treatment include limited access and number of TBI experts for consultation or treatment, lack of a coordinated approach among multiple providers, long travel distances to reach providers, financial barriers, cognitive and physical impairments, and social support limitations (11–15). The ability to address chronic pain and common comorbidities from an accessible, patient centered, coordinated multidisciplinary model is a crucial component of effective pain management and can reduce pain interference in people living with TBI. Services may be delivered by way of flexible, low cost, reliable and accessible methods using telehealth visits supporting an efficient method of delivering health care with regards to time, space, convenience, and cost. Telephone-delivered care has been very well received and effective in our prior studies (16–20).

Collaborative Care (CC) is a patient-centered, team-based approach to delivering evidence-based care. CC approaches include monitoring outcomes, delivering multimodal care, coordinating services, and providing proactive outreach to engage, activate, and promote self-management of symptoms and treatment adherence toward specified targets. Multiple high quality studies and systematic reviews indicate that CC is an effective and sustainable approach to treating depression, chronic pain conditions, and other chronic medical conditions in both primary and specialty care (21–25). Several rigorous clinical trials suggest that it is beneficial in chronic pain conditions within rehabilitation populations (26–28).

This paper describes a collaborative care intervention adapted specifically to treat chronic pain in people living with TBI as part of randomized controlled trial (28). This version of a CC approach, called TBI Care, provided treatment of chronic pain by emphasizing evidence based Cognitive Behavioral Therapy (CBT) for pain and other non-pharmacological approaches into the system of care. Emphasis included options for self-management skills such as relaxation, cognitive reframing, and behavioral activation that were derived from research supporting CBT as an efficacious treatment for chronic pain (29, 30).

The manuscript provides descriptive analysis of the delivery of TBI Care as well as feedback from participants and providers. Additional information regarding study design and primary outcome analysis are available elsewhere (28).

## Methods

### Study setting

TBI Care participants were enrolled from two hospital-based academic outpatient rehabilitation medicine clinics at University of Washington Medical Center – Montlake and Harborview Medical Center. Participants with physician diagnosed TBI of any severity, who endorsed current chronic pain which lasted at least 6 months and were interested in additional assistance for their chronic pain were enrolled from July 2018 through April 2021.

### Participants

79 participants (see Table 1), from a total pool of 158 enrolled participants, who were randomly assigned to TBI Care as part of a randomized control trial in which the other half of participants were assigned to usual rehabilitation care. Eligible clinic participants had to be 18 years or older, have a diagnosis of TBI in their medical record, reported experiencing non-cancer, chronic pain for the last 6 months, had an appointment with their rehabilitation provider, were willing to accept help with their pain, able to read and speak English, had access to phone, and provided informed consent.

**Table 1:** TBI care intervention patient characteristics.

Variables	*N* = 79
Age	
Mean (SD)	47.1 (13.2)
Min, Max	20.5–76.5
Years since injury	
Mean (SD)	3.2 (4.2)
Min, Max	0.5–18.2
Sex	
Male	34 (43%)
Race	
Caucasian (White)	60 (77%)
Asian	7 (9%)
Black or African-American	5 (6%)
American Indian/Alaskan Native	0 (0%)
Native Hawaiian/Pacific Islander	0 (0%)
More than one race	2 (3%)
Other	4 (5%)
Unknown	1
Hispanic	
Hispanic/Latino	5 (6%)
Unknown	2
Marital Status	
Never Married/Single	15 (19%)
Married/Domestic partnership	41 (53%)
Separated/Divorced/Widowed	22 (29%)
Unknown	1
Education years	
Mean (SD)	15.4 (3.0)
Min, Max	6–24
Unknown	5
Employment status	
Working/Student	24 (31%)
Unemployed, looking for work	13 (17%)
Retired/Disabled/On leave	31 (40%)
Other	10 (13%)
Unknown	1
Injury severity	
Mild/Complicated mild	57 (75%)
Moderate/Severe	19 (25%)
Unknown	3

In addition, six UW Medicine TBI Clinic Physical Medicine and Rehabilitation providers were invited to complete a confidential feedback survey.

### Measures

#### Within intervention measures

Treatment response to TBI Care was monitored weekly by the CM over the course of the 12-session intervention. At each session participants were asked to rate their pain intensity (0 = no pain, 10 = pain “as bad as you can imagine”) and interference of pain in two domains; day-to-day activities and enjoyment of life (1 = not at all, 5 = very much), with a goal of 2 or lower on both pain interference items (primary outcome). At every 4th session (sessions 1, 4, 7 & 10), the CM assessed for symptoms of depression, anxiety, and sleep difficulties to monitor change in co-occurring symptoms. Symptoms of depression were assessed using the Patient Health Questionnaire (PHQ)-2 or -9 (31), symptoms of anxiety were assessed using the General Anxiety Disorder (GAD)-2 or -7 (32), and sleep was monitored based on response to the sleep item on the PHQ-9.

#### Post intervention participant feedback

Participants were asked for feedback about the TBI Care intervention by the CM at their last session and at their check in, booster session 2 months later. Participants were asked what components of TBI Care they found helpful and what parts they did not find helpful. At the booster session they were also asked what skills and strategies they were continuing to use. Feedback was acquired through interview, which was written down as it was being provided and then put into the access database under session notes. The database was subsequently reviewed, and each participant's feedback was documented in an excel spreadsheet which was then analyzed for themes.

#### Provider feedback survey

Survey was sent to six providers via Research Electronic Data Capture (REDCap) (33), a secure, HIPAA-compliant, password protected data capture platform hosted by UW, after the intervention. Providers were asked to rate on a 5 point likert scale questions such as: “To what extent do you feel we enrolled those patients who you think needed the intervention?”; “How effective was the communication between you and our CM on the Collaborative Care team?”; “Did being part of collaborative care improve your patient's access to care?”; “How helpful were the recommendations from the collaborative care team (typically conveyed to you by the CM) regarding medications or other referrals?”; “Did you see improvement in the following areas (pain lowered; mood improved and/or anxiety lessened; sleep improved; physical activity improved; coping strategies improved)?” They were also asked, “Among your patients who were enrolled in collaborative care arm, do you think they had a better, worse, or similar outcome after working with the CM?”

### Study intervention: TBI care collaborative care intervention

**Figure 1:**
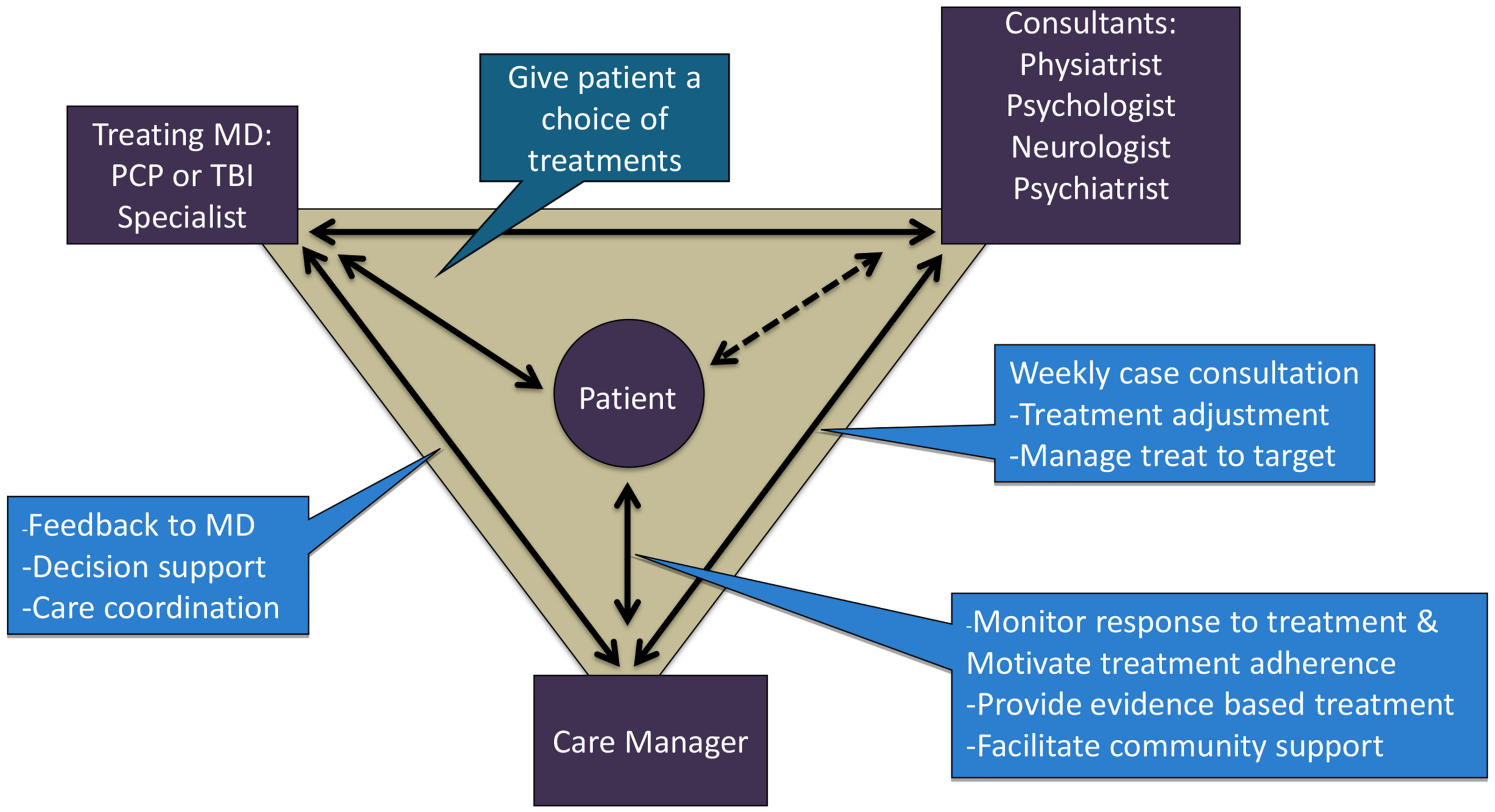
Collaborative care model.

Based upon the core principles of CC (34), TBI care was structured around the participant who worked with a team (see Figure 1) made up of the CM (M.C.), the expert team comprised of a rehabilitation psychologist (J.H.), physiatrist (J.Z.), psychiatrist (J.R.F.) and headache specialty neurologist (S.L.), and the treating clinician (i.e., rehabilitation medicine clinic provider).

The CM offered and scheduled up to 12 sessions over 16 weeks with each participant and communicated via notes in the electronic health record (EHR) and as needed. The CM met for an hour weekly with the expert team to review all active participants, with a focus on those who were not improving or had challenges with current treatment (e.g., tolerability, adherence). The expert team offered recommendations related to treatment (e.g., referrals, exercise considerations, non-pharmacologic treatment to consider) and suggested medication changes (e.g., dose/timing changes, starting new medications, tapering off medications).

Consistent with the CC model, each session included a review of the participant's clinical status, progress from prior sessions and review of medications. In TBI Care, we then focused on CBT interventions to address current symptoms and modified based on cognitive or other difficulties related to TBI. The CM checked on participant medication adherence and any changes at each session and reviewed with the team as needed at weekly case consultation. CC traditionally uses clinical practice guidelines (CPGs) to guide medication management, however, there are no CPGs for pain management after TBI. The expert team utilized CPGs from other disease states (non-TBI populations) (35) and modified based on knowledge of the current TBI literature as well as their clinical experience with TBI. The weekly consult focused on personalized and intensive treatment recommendations to address the specific needs of the participant.

To the best of our knowledge, TBI Care differed from most other collaborative care models in the literature in that it included a rehabilitation psychologist and physical medicine and rehabilitation physician (physiatrist) as domain experts who provided TBI expertise for addressing pain management in this population. The CM set the agenda ahead of time for weekly consult meetings, allowing for a more strategic use of expertise and resources based on current participant needs.

**Figure 2:**
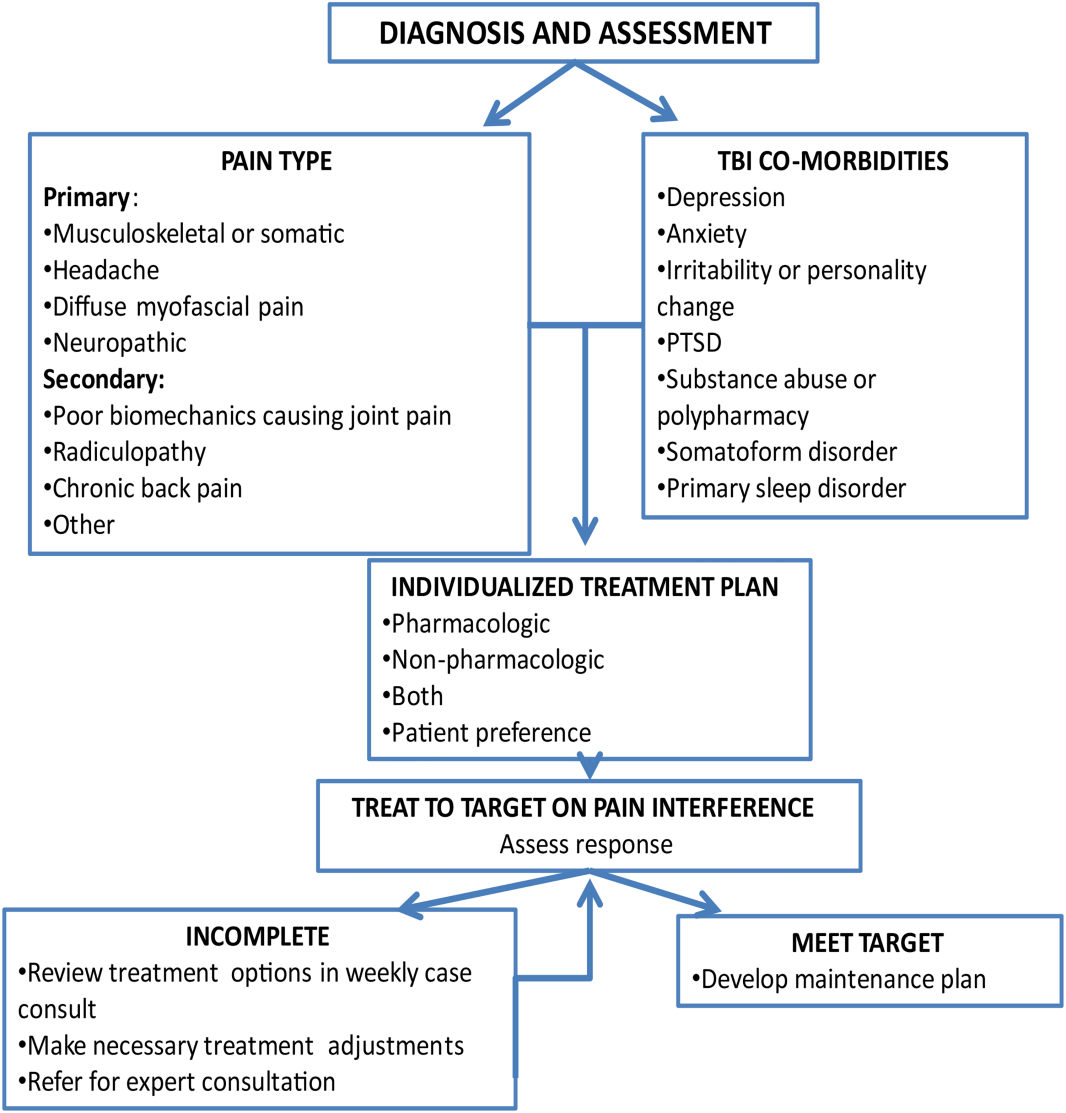
Flow of TBI Care Intervention.

The flow of the intervention (see Figure 2) was individually tailored to participant needs, goals and the type(s) of pain they endorsed.­ The CM and expert team reviewed the EHR to gather information on diagnoses, comorbid conditions, prior treatments, and response to treatment along with information obtained at screening. As part of the first session, the CM reviewed options for individualized participant pain treatment, including treatments currently recommended by their treating physician. The options included adding non-pharmacologic treatment or adjustment of pharmacologic treatment or both depending on the participant's preference.

A key ingredient to CC is outreach, to ensure that participants are not lost to follow up. Thus, the CM provided proactive and consistent outreach including treatment reminders, reaching out to those who missed sessions, and following up on treatment recommendations via participants preferred method of contact (text, phone, or email).

The initial session focused on engagement, building therapeutic alliance, orienting to TBI Care, and creating a patient centered preliminary treatment plan. This was done through providing an overview of the program, pain psychology education, highlighting potential benefits of treatment, a structured clinical assessment of current pain and medications, treatment history, and joint decision making on treatment goals & planning.

**Figure 3:**
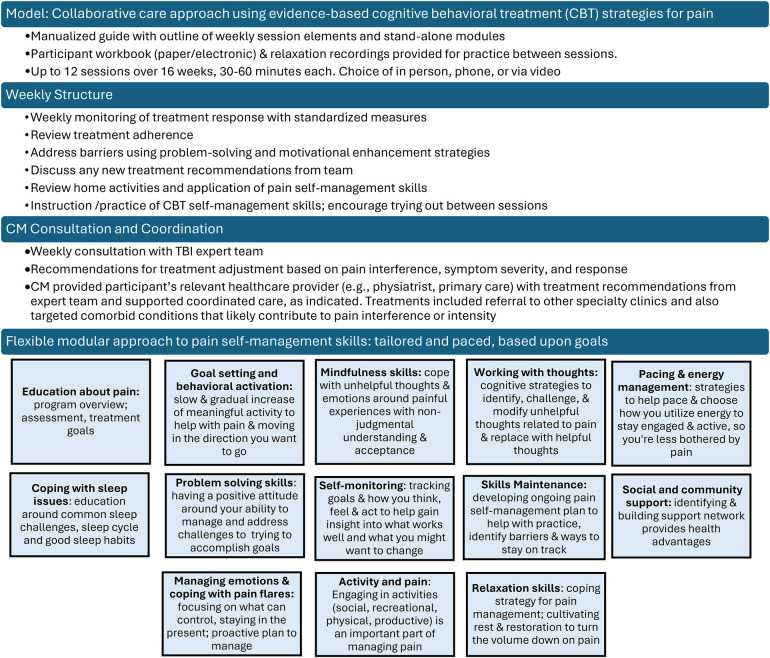
Overview of TBI care collaborative care intervention.

The CM followed a similar format for each session (see Figure 3). The CM monitored pain intensity and interference weekly, and depression, anxiety, and sleep symptoms every 4th session or as indicated. After each session, a brief session summary was documented in the participant's EHR, CM copied the attending PMR provider and completed any necessary follow-up and care coordination.

The last session (typically session 12) focused on reviewing skills, skills maintenance (e.g., identifying barriers to skill use and ways to stay on track) and developing a plan to maintain skills and continue with other treatment and recommendations. The skills maintenance plan was sent to the participant and documented in their EHR. A check-in call (20–30 min) was conducted 8 weeks following the last session to discuss how the participant was doing since ending treatment. The focus of the check-in call was to see if they needed assistance or had questions post intervention. This included a review of symptoms in which pain intensity & pain interference were monitored along with depression, anxiety and sleep symptoms, reinforcement of maintaining skills learned in treatment, strategies for getting back on track and recommendations for further treatment and self-management strategies**.**

The goal of the intervention was to identify individualized recommendations that met a participant's stated needs and preferences, including where to focus the cognitive behavioral skill building, additional non-pharmacological treatments to consider, and medication recommendations for pain treatment according to the pain and/or headache type, and presence of comorbid conditions. Given the specific needs of people living with TBI, the team would typically recommend a medication that could both treat pain and a co-occurring condition while exercising caution around medications that may cause significant cognitive side effects.

Care coordination was facilitated by the CM who collaboratively discussed the follow up plan with the participant, identifying agreed upon next step responsibilities, with an emphasis on participant responsibility. The CM then circled back to the participant to ensure action steps happened or helped to problem solve barriers. This allowed participants to identify what works best for them, as there were participants who could follow through on their own, and others who benefitted from the CM talking with providers directly.

An important advantage and key component to CC is the ability to modify or intensify care in a timely way when participants are not responding to their treatment. The TBI Care team reviewed participant self-reported outcomes (treating to primary target of pain interference) from each session and treatment recommendations were systematically intensified or modified if needed (e.g., decreasing medication triggers of rebound headaches) to assist in improving the participant's response.

The CM offered relevant patient education, utilized strategies to engage, motivate and enhance adherence to the medical treatments (including community- or home-based physical activity), and provided a person-centered modular approach of evidence-based CBT to target both pain and comorbid conditions (see Figure 3). Module content addressed the specific challenges of persons living with TBI, with the flexibility to slow down content delivery or repeat material as needed to insure understanding and relevance to the direct life experience of participants.

### TBI care CM training

The CM was a clinical social worker with previous experience working in CC and providing CBT treatment for persons living with disability, chronic pain, and depression. The CM received formal training as needed by co-investigators with relevant experience in treating pain in persons living with TBI, particularly headache, and reviewed specific study procedures and the utilization of the collaborative care model for this population. Didactic training consisted of lectures, experiential training, and online resources. Ongoing training opportunities for the CM occurred throughout the intervention as needed.

The CM met with clinic staff and providers prior to launching the intervention to learn more about clinic flow, systems, and get their input on how best to coordinate care (e.g., sleep medication follow-up may be with the clinic rehabilitation nurse or the CM for a participant in TBI Care). The training also involved shadowing providers at both clinic sites to gain perspective on common needs of people living with TBI and chronic pain.

### Fidelity monitoring

Weekly individual consultation provided monitoring of fidelity to the CC model through session review. We utilized both a summary of each participant as well as a review of data collection (e.g., standardized measures, content covered). In this case review process, one consultant (JMH) reviewed the intervention database to assess recorded session content and added relevant information related to consultation recommendations and plan for follow up.

## Results

Of the 79 participants randomized to TBI Care, 78 opted to engage in the intervention. Participants were offered up to 12 sessions and 90% of participants received 11–12 sessions. To help reduce barriers to treatment, flexibility was built into the model and participants were offered a choice of receiving sessions in person, over the phone or via HIPPA compliant video, or a combination of all three depending on participant preference. Of the 78 participants who completed at least 1 session, 62 received one or more sessions over the phone (62% of total sessions), 30 participants had 1 or more in-person sessions (accounting for 12% of the total sessions) and 25 had one or more video sessions (accounting for 26% of sessions). Due to the COVID-19 pandemic, beginning mid-March of 2020 all sessions took place remotely, either over the phone or via video. Thirty-six participants (46%) completed their last session pre-COVID, with 42 participants (54%) completed during COVID-19, the last of whom finished in August of 2021. Emergency COVID-19 orders in Washington state continued through October 31, 2022 (36).

### Fidelity to the intervention

**Figure 4:**
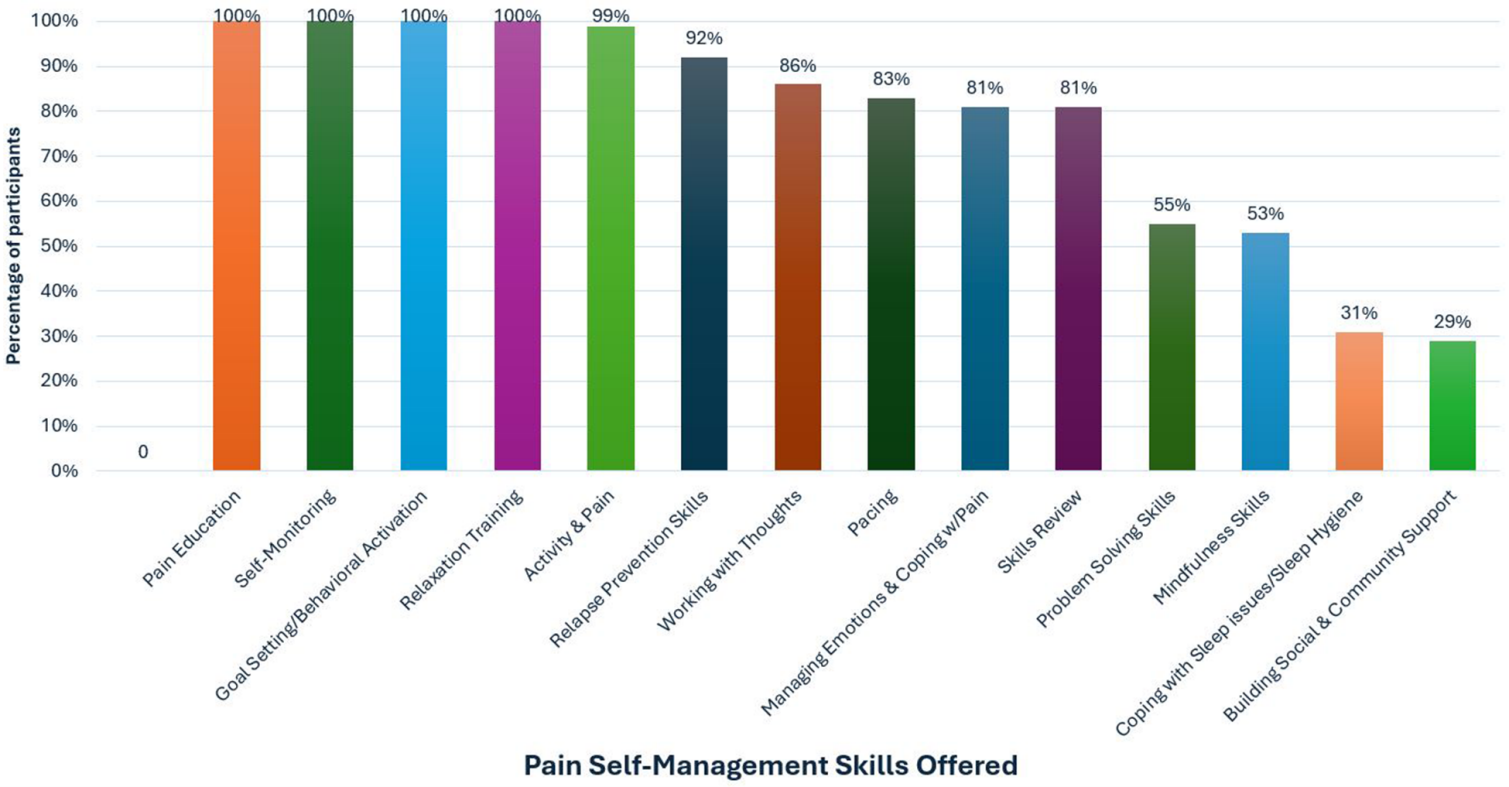
Pain self-management skill modules received.

Session content was guided using a person-centered approach in which we adjusted modules on evidence-based cognitive behavioral skills and pace of the intervention based on participant need and preference. All participants received modules on TBI Care study overview, pain psychology education, self-monitoring, goal setting, behavioral activation, and relaxation training (see Figure 4). Figure 4 provides an overview of the proportion of participants who received each skill throughout TBI Care.

### Treatment & medications recommendations over the course of the intervention

At weekly consult meetings, the CM reviewed with the team the CBT skills focused on with each participant during the previous session. Discussion with the expert group included suggestions for building upon, reinforcing, or adding additional CBT skills for pain management for the majority (89%) of participants, while 65% were offered recommendation or referral for treatment or encouraged to connect with their provider around treatment options and 43% of recommendations focused on care coordination.

Recommendations related to medication included: considering a new medication (47%), dose change (34%), providing information on how to take (34%), and responding to questions around impact of a new medication or change in dose. Of the 37 participants (47%) who received a recommendation for a new medication during treatment, the majority targeted headache (59%). The CM checked in verbally with participants on medication adherence and any changes at each session and reviewed with the team as needed at weekly consult. TBI Care participants reported being on slightly more medications and supplements (prescribed, over the counter, & vitamins/supplements) at the last session of treatment than the first (6.9 vs. 8.2) with nearly 75% of participants reporting being on 6 or more medications and supplements at both the first and last session. No participant reported being on zero medications during the intervention (see Table 2).

**Table 2:** Summary of medication/supplement usage.

	First session	Last session
TBI Care participants	79	79
Number of medications		
Mean (SD)	6.9 (3.5)	8.2 (3.4)
Median (IQR)	6.5 (4.25, 9)	8 (6, 10)
Min, Max	1, 17	1, 19
0	0 (0%)	0 (0%)
1–5	20 (26%)	20 (26%)
6+	58 (74%)	58 (74%)
Unknown	1	1

### Participant within intervention measures

**Figure 5:**
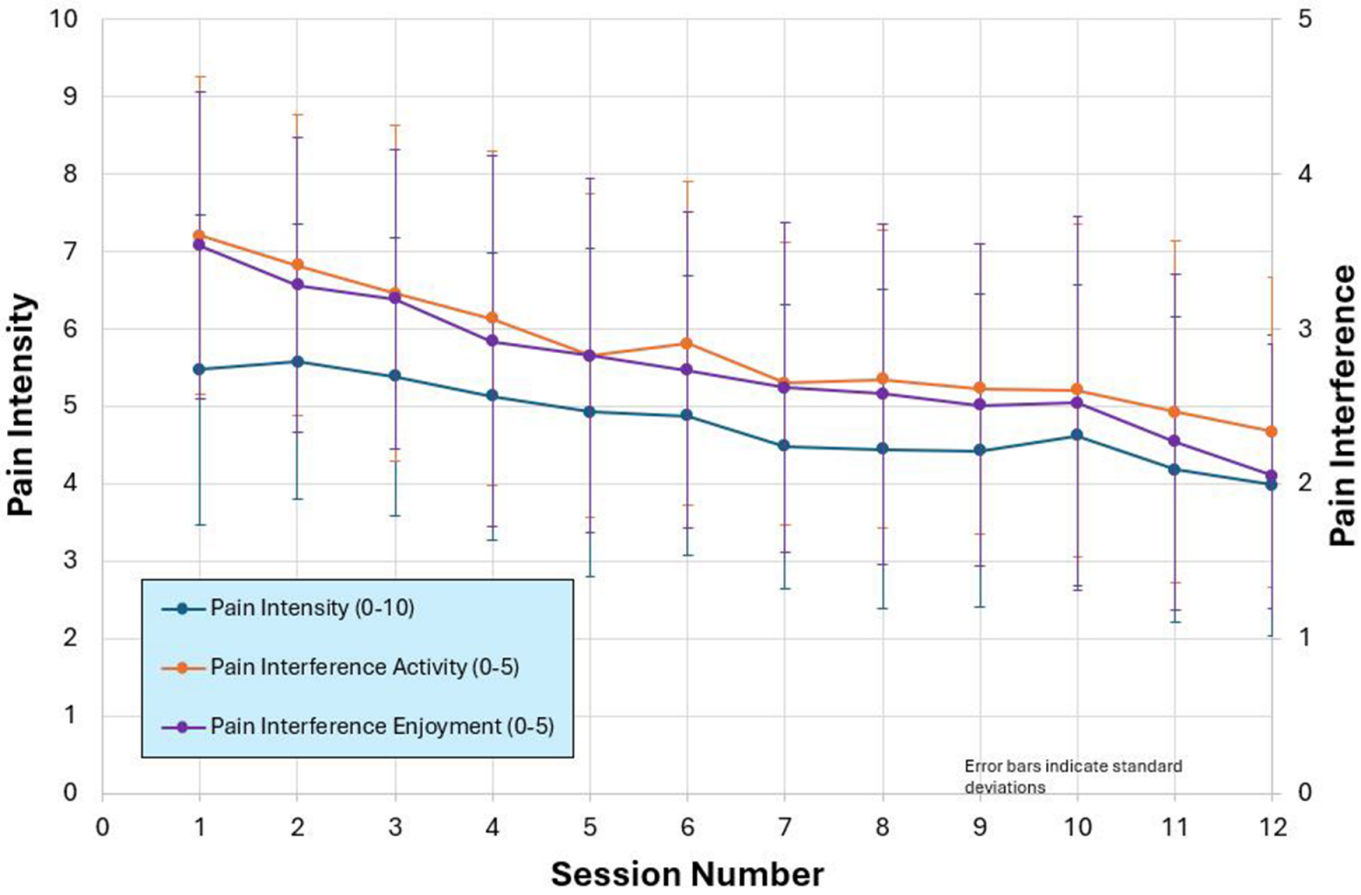
Pain intensity and interference ratings over the course of treatment.

TBI Care participants self-reported a decrease in pain interference (1 = not at all, 5 = very much) in activity (3.5 to 2.4) and enjoyment of life (3.5 to 2) and pain intensity (0 = no pain, 10 = pain as bad as you can imagine; 5.5 to 4) (see Figure 5). In addition, the percentage of participants endorsing the cardinal symptoms of depression (feeling down, depressed, or hopeless and/or lack of interest or pleasure in doing things) on the PHQ-2, reduced from 35% at Session 1% to 21% by Session 10. The percentage of participants endorsing anxiety symptoms per GAD-2 (first 2 items of the GAD-7), feeling nervous, anxious or on edge and/or not being able to stop or control worrying), went from 45% to 33%.

### Participant feedback about treatment

Themes emerged highlighting satisfaction with treatment, skill building and the CC model of care. As one participant stated, “Being part of TBI Care helped by having structure, tools, workbook and information to refer to & I felt like I was not alone; I appreciated adjusting modules for my needs & being able to talk over phone made it easier; doing the logs every day was useful.” Table 3 includes example quotes from participants on the connection between their emotions and pain, the skills they learned and how helpful they found the CC approach. For example, one participant expressed “…holistic approach was important, skills invaluable and very effective, before TBI Care I felt like I was on my own, it helped me figure out and come up with coping mechanisms; having more tools and understanding, has helped with pain management; so pain way more manageable.”

**Table 3:** TBI care participant feedback.

Participant feedback around connections between thoughts/emotions & pain: • TBI Care “changed the way I look at things, didn't realize how negative my thought process was” & learned that “pain was consuming me, then I ignored everything, now know that I have tools I can use.” • Pt says that the information/skills provided by TBI Care around how thoughts impact pain “incredibly beneficial.” Pt reports being part of TBI care study has helped to shift what pt tells self re: pain, now says “going to live life despite pain.” • “When I started the study I didn't think taking time away from things was an option, now know it is easy to take time; also mentally recognizing when not feeling well, look at situations objectively, & remind self no need to be frantic or worried.” Pt says found “focusing on what has control over” as part of TBI care helpful and continues to use that as a strategy.
Skill Building & learning about pain management • “I didn't get info about pain management in the beginning; it was all kind of new, scary; having the workbook and talking w/ someone was more reassuring, knowing that there are things I can do to ease pain a little bit; that I have skills to help.” • “Knowing how much pain can affect a person with TBI; pacing element huge”; “daily and weekly logging & setting achievable goals on paper is so helpful as is the breathing.”
Collaborative care helpful • Collaborate care team helped, “having multiple people looking at my story”; & “having a half hour each week to sit down, focus on one thing and realized how I am doing; plans to keep doing this.” • “TBI Care helped provide useful tools” and collaborative care helped “to keep moving things forward.” • Following parts of TBI care helpful: “care coordination & accountability; reinforced that I was doing the right things; helped to stay motivated and learning relaxation skills.” Pt says that “learned as part of TBI Care to use multiple tools” & states “TBI care helpful around the accountability & encouraging me to follow up with care.” • “Care coordination, checking in on meds so helpful, helped to look at big picture; breathing exercises and support for taking care of myself.” Pt states the following skills from TBI care helpful & have stuck: “really checking in with myself, planning schedule/days ahead of time and honoring my breaks; & catching myself early when I overdo.”

### Provider feedback

Overall, 5 of the 6 providers completed the survey and reported that they were satisfied with TBI Care. 80% felt it improved their patient's access to care either quite a bit or very much, while the majority indicated the amount of time they or the clinic needed to spend with TBI Care patients lessened. All felt their patients involved in TBI Care had better outcomes, noting improvements in physical activity, mood, sleep, and coping strategies, with pain lowering somewhat. A number felt that the regular check-ins provided by CM were noticeable and demonstrated their importance. One provider commented, “Patients were receiving frequent education on pacing, anxiety/relaxation techniques, goal setting etc. through the study, so I did not need to spend as much time discussing these items during our visits.”

## Discussion

This paper describes a collaborative care model adapted specifically for chronic pain in persons living with TBI. The participants were complicated in that they were an average 3 years out from their injury, had multiple pain locations, and virtually all had headache pain that interfered with their function. The majority were on an average of 6 or more medications and supplements during the intervention and many reported significant symptoms of depression (35%) and anxiety (45%). Despite this complex group, participants showed a decrease in pain intensity and interference throughout the course of treatment and reported high rates of satisfaction with care. Indeed, 90% were committed to the intervention, attending 11 or 12 of the scheduled 12 sessions, allowing for maximal engagement with the CM.

Our approach to collaborative care was novel in that it was a patient-centered, biopsychosocial approach that leveraged a multidisciplinary team of expert consultants that could address the complex nature of TBI-related pain and its common comorbidities. We designed our primary intervention with the focus on CBT for chronic pain. The model relied on the participant being an active member of the team. It utilized core principles and structures of collaborative care, adding subspecialists appropriate to the nature of the participant's pain.

The engagement of participants may be related to the fact that the CM ensured the intervention was flexibly delivered and tailored to the unique needs of people living with TBI. The emphasis was on matching the pace of the sessions to the needs of the participant, especially when cognitive challenges were present. This flexibility (e.g., offering telehealth visits, slowing down delivery of content, or repeating to help with retention and understanding) was an important piece of optimizing care and ensuring the intervention was relevant to the direct experience of people with TBI. This meant that certain modules were not received by all participants, even though they may have provided benefit. We focused on helping participants develop a solid set of skills meaningful to them, while giving them a workbook covering all skills (see Figure 4) to refer to if they chose to access additional skills at a later date. Similar strategies have been used in prior CBT interventions in TBI (19, 37). We used our expert provider input to facilitate the use of strategies and treatments for chronic pain that are considered evidence based-treatments in other populations with chronic pain (38).

Finally, the feedback from participants and providers is consistent with prior research. In two prior CC studies in rehabilitation medicine clinics, participants endorsed higher satisfaction with health care (26, 39). Additionally the larger literature on CC highlights that patient and care providers express an overall positive experience with CC, with patients noting it as helpful in their recovery and that the model was acceptable (40), as well as patients & providers citing benefits around accessibility and referral process (41). However, while providers see CC as a way to improve the management of depression, there are barriers to implementation (42). Therefore, future work should include implementation sciences approaches to improve implementation and dissemination of this model.

## Study limitations

This study was limited to English speaking adults at least 6 months post TBI with chronic pain who were outpatients at two TBI clinics and who were willing to receive additional help with their pain. These findings may not represent individuals with TBI in the acute phase of recovery or those not receiving outpatient care in Rehabilitation Medicine clinics. Consistent with pain and psychosocial research we used self-reported measures, which may have some level of bias due to inaccurate recall, and/or cognitive challenges. Medication recommendations were provided throughout the intervention, though the CM checked in on medication adherence at each session (e.g., whether a participant started, stopped, or had a dose change with a medication), we were not able to track how many participants followed through on these recommendations over time.

## Conclusion

Using a CC model in TBI was feasible, effective, and improved access to evidence-based non-pharmacological and pharmacological treatments for chronic pain. The TBI Care intervention resulted in significantly lower pain interference & pain intensity. Intervention participants were highly engaged in treatment and expressed a benefit from this flexible patient centered model of care that provided an accessible, multifaceted, and holistic (looking at one's physical, emotional, and social well-being) approach to pain management. In addition, providers expressed high satisfaction with this model. TBI Care is a promising approach to treat chronic pain in individuals with TBI. Future research is needed to study the model's cost-effectiveness, implementation, and scalability in diverse healthcare settings. In addition, we would like to see further research to determine expanding this model to veterans and those impacted by the polytrauma clinical triad (43) of post-traumatic stress disorder, chronic pain and TBI.

## Data Availability

The original contributions presented in the study are included in the article/Supplementary Material, further inquiries can be directed to the corresponding author.
